# Quantifying Glial-Glial Tiling Using Automated Image Analysis in *Drosophila*

**DOI:** 10.3389/fncel.2022.826483

**Published:** 2022-03-24

**Authors:** Gabriela Salazar, Grace Ross, Ariana E. Maserejian, Jaeda Coutinho-Budd

**Affiliations:** ^1^Department of Biology, The University of Vermont, Burlington, VT, United States; ^2^Vermont Complex Systems Center, The University of Vermont, Burlington, VT, United States

**Keywords:** glia, tiling, *Drosophila*, morphology, astrocytes, automated image analysis, cortex glia, glial-glial interaction

## Abstract

Not only do glia form close associations with neurons throughout the central nervous system (CNS), but glial cells also interact closely with other glial cells. As these cells mature, they undergo a phenomenon known as glial tiling, where they grow to abut one another, often without invading each other’s boundaries. Glial tiling occurs throughout the animal kingdom, from fruit flies to humans; however, not much is known about the glial-glial interactions that lead to and maintain this tiling. *Drosophila* provide a strong model to investigate glial-glial tiling, where tiling occurs both among individual glial cells of the same subtype, as well as between those of different subtypes. Furthermore, the spatial segregation of the CNS allows for the unique ability to visualize and manipulate inter-subtype interactions. Previous work in *Drosophila* has suggested an interaction between cortex glia and astrocytes, where astrocytes cross the normal neuropil-cortex boundary in response to dysfunctional cortex glia. Here, we further explore this interaction by implementing an automated pipeline to more fully characterize this astrocyte-cortex glial relationship. By quantifying and correlating the extent of cortex glial dysfunction and aberrant astrocyte infiltration using automated analysis, we maximize the size of the quantified dataset to reveal subtle patterns in astrocyte-cortex glial interactions. We provide a guide for creating and validating a fully-automated image analysis pipeline for exploring these interactions, and implement this pipeline to describe a significant correlation between cortex glial dysfunction and aberrant astrocyte infiltration, as well as demonstrate variations in their relationship across different regions of the CNS.

## Introduction

Neurons and glia comprise the majority of the cells in the central nervous system (CNS). We often think of neurons as having the main function—signal transmission—whereas glia perform a variety of supportive duties. Glia sculpt neurons during development and plasticity (Spindler et al., 2009; Schafer et al., 2012; Tasdemir-Yilmaz and Freeman, 2014; Gunner et al., 2019), engulf debris in development, injury, or disease (MacDonald et al., 2006; Hong et al., 2016; McLaughlin et al., 2019; Werneburg et al., 2020), provide neurons with key nutrients and metabolic support (Spindler et al., 2009; Volkenhoff et al., 2015; Fernandes et al., 2017; Ioannou et al., 2019; Li et al., 2020), ensheath axons for proper axonal conduction and integrity (Kottmeier et al., 2020; Madden et al., 2021; Nave and Werner, 2021), maintain the blood brain barrier (Bainton et al., 2005; Schwabe et al., 2005; Stork et al., 2008; Heithoff et al., 2021) and buffer ions and neurotransmitters to modulate neuronal activity (Melom and Littleton, 2013; Muthukumar et al., 2014; Ma et al., 2016). Given the wide range of these and additional functions, it is not surprising that glia have been shown to play roles in a number of neurological disorders such as Autism, Epilepsy, Schizophrenia, as well as neurodegenerative disorders like Alzheimer’s disease (Blanco-Suárez et al., 2017; Salter and Stevens, 2017; Patel et al., 2019; Dietz et al., 2020; Gleichman and Carmichael, 2020). Thus, elucidating glial function is a crucial step in achieving a thorough understanding of the brain.

In addition to interacting extensively with neurons, glia also form complex physical and signaling interactions with each other. One of the ways in which glial-glial interactions manifest is a phenomenon known as tiling, where each glial cell grows to fill a space without invading the boundaries of others. Many glial cells form almost perfectly tiled domains that exhibit very little overlap between cells; however, the exact amount of overlap between these glial domains can vary between species, age, and disease state (Oberheim et al., 2008, 2009; Grosche et al., 2013; López-Hidalgo et al., 2016). Tiling can be observed between glia of the same subtype, such as between two or more astrocytes (Bushong et al., 2002; Stork et al., 2014; Chen et al., 2020), microglia (Kettenmann et al., 2013), oligodendrocyte precursor cells (Hughes et al., 2013), Müller glia (Wang et al., 2016) as a few examples, as well as between glia of different subtypes (Stork et al., 2008; Coutinho-Budd et al., 2017; Kremer et al., 2017). Importantly, glial tiling and domain organization is highly conserved among species from flies to humans (Oberheim et al., 2009; Kremer et al., 2017); however, little is known about the interactions that lead to and maintain glial tiling in any species. Moreover, the functional relevance of this tiling remains almost completely unexplored.

**FIGURE 1:**
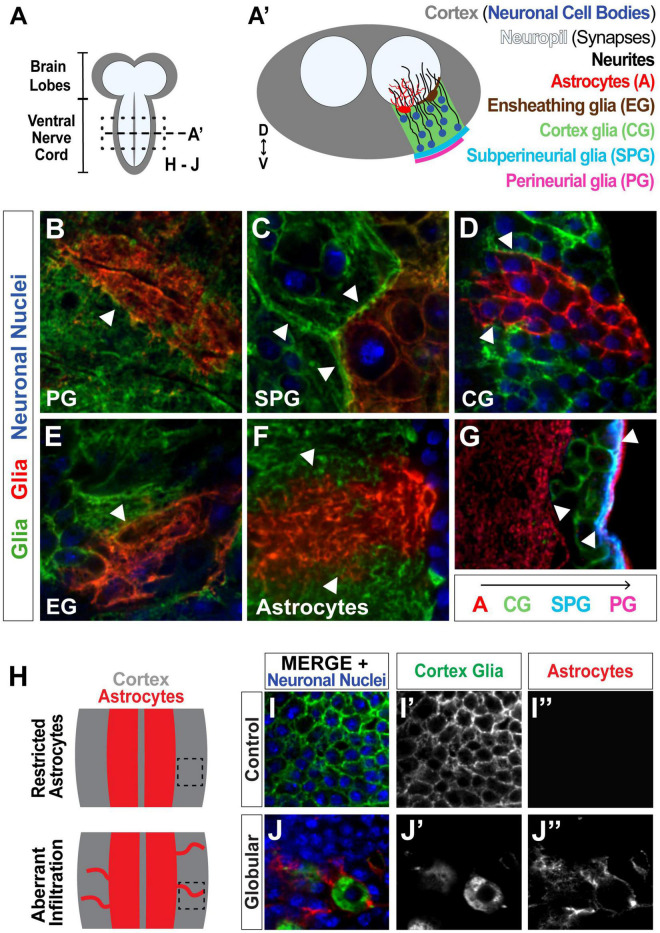
*Drosophila* serves as a strong model for investigating glial tiling. **(A)** Schematic of the *Drosophila melanogaster* larval central nervous system (CNS), subdivided into the cortex (gray, containing the cell bodies) and the synaptic neuropil (white). **(A’)** Cross-section of the ventral nerve cord (VNC) depicting neuronal nuclei (blue) and single cell examples of different glial subtypes to show their distinct spatial segregation: Perineurial glia (PG, magenta) and subperineurial glia (SPG, cyan) form a barrier around the CNS, cortex glia (CG, green) wrap neuronal cell bodies, and ensheathing glia (EG, brown) and astrocytes (red) are associated with the neuropil. **(B–F)** Tiling between cells of the same glial subtype shown in green and red with neuronal nuclei shown in blue: PG (**B**, surface view), SPG (**C**, surface view), CG **(D)**, EG (**E**, surface view), astrocytes **(F)**. **(G)** Tiling between different glial subtypes: PG (magenta), SPG (blue), CG (green), astrocytes (red). Examples of tiling boundaries depicted with white arrowheads. **(H)** Schematic illustrating astrocyte aberrant infiltration (red) into the cortex (gray) upon disruption of CG morphology. **(I,J)** Images corresponding to black inset in **(H)**, showing control **(I)** and globular **(J)** cortex glial conditions, where astrocytes can be seen infiltrating the cortex in **(J”)**. Cortex glia in green **(I’,J’)**, astrocytes in red **(I”,J”)**, neuronal nuclei in blue.

As a model, *Drosophila melanogaster* strikes a balance between simplicity and complexity that makes it especially suitable for conducting a thorough examination of glia-glial interactions. Despite its simplicity, the fly CNS maintains a high degree of complexity, composed of multiple neuronal and glial subtypes that share cellular, genetic, and functional conservation with their mammalian counterparts (Awasaki et al., 2008; Yildirim et al., 2019). Furthermore, because of the high level of genetic, proteomic, signaling, and cellular conservation from flies to mammals, many findings made using *Drosophila* are applicable to understanding the mammalian brain (Bellen et al., 2010). The elegance of *Drosophila* genetics has allowed for the development of a vast and powerful arsenal of genetic tools that makes this an attractive model for investigating glial tiling (Bellen et al., 2010; Awasaki and Lee, 2011; Rodríguez et al., 2012). Specifically, these tools allow for genetic labeling and manipulation of either single cells or entire cell-type populations, as well as applying different genetic alterations to multiple cell types at the same time (Figure 1).

The spatial organization of the *Drosophila* CNS makes this a particularly ideal model for studying glial-glial tiling (Figures 1A,A’). The *Drosophila* CNS is subdivided into two main regions, the cortex and neuropil, where the cortex comprises the neuronal cell bodies and the neuropil contains the synapses. Furthermore, the CNS contains multiple glial cell types that spatially segregate along these regions, and are known to tile within and between subtypes. Perineurial glia (PG, Figure 1A’, surface view in Figure 1B), and subperineurial glia (SPG, Figure 1A’, surface view in Figure 1C) wrap the CNS to form the blood-brain barrier (Bainton et al., 2005; Schwabe et al., 2005; Awasaki et al., 2008; Stork et al., 2008; Hartenstein, 2011). Cortex glia (CG, Figures 1A’,D) intersperse among the neuronal cell bodies in a mesh-like pattern to wrap and support the somas (Awasaki et al., 2008; Hartenstein, 2011), where each cortex glial cell wraps 50–100 neuronal cell bodies (Coutinho-Budd et al., 2017; Kremer et al., 2017), providing metabolic support (Volkenhoff et al., 2015), and debris clearance in the cortex (Coutinho-Budd et al., 2017; McLaughlin et al., 2019). Ensheathing glia (EG, Figure 1A’, surface view in Figure 1E) and astrocyte cell bodies are located on the interface between the cortex and neuropil (Awasaki et al., 2008; Hartenstein, 2011), where EG processes form a barrier between the two regions (Pogodalla et al., 2021), and astrocytes extend fine processes into the neuropil (Figures 1A’,F) that interact with synapses (Muthukumar et al., 2014; Stork et al., 2014; Tasdemir-Yilmaz and Freeman, 2014; Ma et al., 2016). Each of these subtypes forms tight boundaries between cells of their own kind (Stork et al., 2014; Coutinho-Budd et al., 2017; Kremer et al., 2017; Pogodalla et al., 2021), as well as between different glial subtypes such as astrocytes and cortex glia, cortex glia and SPG, or SPG and PG (Figures 1A’,G; Stork et al., 2008; Coutinho-Budd et al., 2017; Kremer et al., 2017). We have previously shown that upon morphological disruption of cortex glia caused by the loss of the neurotrophin spätzle 3 (Spz3) or soluble NSF attachment protein α (αSNAP, part of the vesicular fusion machinery the vesicular fusion machinery that leads to Spz3 secretion), neuronal cell bodies lose their physical interactions with cortex glia, and astrocytes extend aberrant processes into the cortex (Figures 1H–J; Coutinho-Budd et al., 2017); however, the previous report found that this phenomenon occurs, but did not quantify the extent to which it occurs or the relationship between the degree of cortex glial morphological disruption and aberrant astrocyte outgrowth.

In an effort to more fully characterize the tiling relationship between cortex glia and astrocytes, and to further establish this model for investigating the disruption of glial-glial tiling, we sought to implement an automated analysis of the extent of globular morphological transformation of cortex glia and astrocyte infiltration, and assess the relationship between the two characteristics. Here we describe a method for creating and validating an automated image analysis pipeline using free, open-source software. Using this optimized pipeline, we reveal a significant correlation between the extent of cortex glial morphological disruption and aberrant astrocyte infiltration. Additionally, these data allow us to explore regional variations in morphology and infiltration throughout the dorsal-ventral axis of the CNS.

## Materials and Tools

### Fly Strains

*Drosophila melanogaster* crosses were raised at 29°C on Nutri-fly Molasses Formulation food (Genesee Scientific). The following previously made transgenes were used in this study: *Wrapper932i-LexA* (Driver 1) (Coutinho-Budd et al., 2017), *CtxGliaSplit-Gal4* (Driver 2) (Coutinho-Budd et al., 2017), *GMR54H02-Gal4* (BDSC 45784), *alrm-Gal4* (with Driver 1) (Doherty et al., 2009), *alrm-LexA::GAD* (with Driver 2) (Stork et al., 2014), *GMR56F03-Gal4* (BDSC 39157), *GMR85G01-Gal4* (BDSC 40436), *GMR54C07-Gal4* (BDSC 50472), *Mi{PT-GFSTF.0}trolMI04580-GFSTF.0* (BDSC 60214), repoFLP, *UAS-CD8*>*GFP*>*RFP* (Stork et al., 2014), *UAS-αSNAP*^RNAi^** (VDRC 101341), *LexAop2-Spz3*^RNAi^** (Coutinho-Budd et al., 2017), *UAS-CD8::GFP* (Lee and Luo, 1999), *UAS-CD8-mCherry* (Stork et al., 2014), *LexAop-rCD2::GFP* (Lai and Lee, 2006), *and LexAop-rCD2::RFP* (Lai and Lee, 2006).

### Immunohistochemistry and Imaging

The larval CNS was dissected in the third instar larval stage. The samples were fixed in ice-cold 100% methanol for 5 min at room temperature, then rinsed three times with PTX (PBS + 0.1% Triton-X). Samples were stained overnight with primary antibodies at 4°C, rinsed three times with PTX, then stained overnight with secondary antibodies at 4°C. The following primary antibodies were used: chicken anti-GFP (1:1000; Aves Labs), rabbit anti-dsRed (1:500; Clontech), rat anti-Elav (1:100; Developmental Studies Hybridoma Bank, 7E8A10), rabbit anti-GAT (1:2000; Stork et al., 2014), rat anti-CD2 (1:500; Bio-Rad). The following secondary antibodies were used: donkey conjugated to DyLight 488 [anti-chicken (103-005-155)], Cy3 [anti-rabbit (711-165-152)], and Cy5 [anti-rat (712-175-150)] from Jackson ImmunoResearch. After washing three times with PTX, samples were mounted in VectaShield reagent (Vector Laboratories) and imaged on an Intelligent Imaging Innovations (3i) spinning disk confocal microscope equipped with a Yokogawa CSX-W1. Finally, out-of-focus images from the beginning or end of the stack were removed. A total of 3,309 images from 84 three-channel confocal Z-stacks marking astrocytes (red channel), cortex glia (green channel), and neuronal cell bodies (blue channel) were analyzed.

### Image Processing and Automated Pipeline Analysis

**FIGURE 2:**
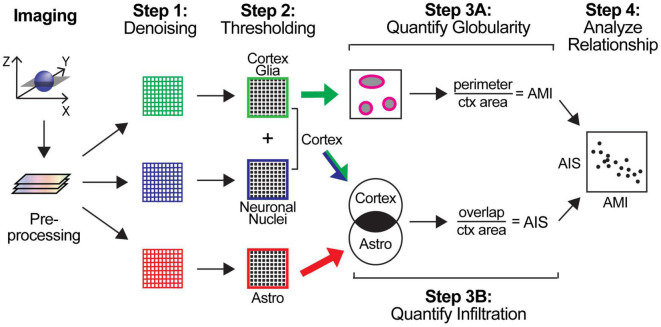
Schematic of automated image analysis pipeline for 3-channel confocal Z-stacks. Preprocessing: Z-stacks were separated into 2D arrays representing a single plane for each cell type (corresponding to a single channel for each). Step 1: Individual channel images were denoised. Step 2: 2D arrays were thresholded to produce binary images. Step 3A: The perimeter of cells in the cortex glia (CG) channel was measured and normalized to total cortex area producing an automated morphology index (AMI) score. Step 3B: CG and neuronal nuclei channels were combined to define the cortex area (ctx). The overlap between the ctx and astrocyte (astro) channels was calculated and normalized to the total cortex area to produce an automated infiltration score (AIS). Step 4: The relationship between the AMI and AIS was analyzed.

This pipeline was specifically designed to take advantage of open-source software that allows for its implementation by nearly anyone with access to two-dimensional images. Importantly, it does not require access to more expensive three-dimensional imaging capability like light sheet microscopy or specialized 3D image analysis software licenses, making this tool easy for almost anyone to implement to maximize their analyses and reduce unintentional bias that can occur with manual quantification. Briefly, following preprocessing, the images were fed into the pipeline, separated into individual single-channel 2D-images and denoised. The images were then thresholded to produce binary images, and simultaneous scoring occurred for both globularity and infiltration. Cell perimeter was used as a proxy for quantifying cortex glia morphology, while aberrant astrocytic process infiltration was quantified by measuring the overlap between the astrocyte channel and the cortex, determined by combining the cortex glial and neuronal channels. Finally, the scores produced by pipeline were analyzed to assess the relationship between cortex glia morphology and aberrant infiltration by astrocytes (Figure 2). Scikit-image (Walt et al., 2014) was used for all automated image processing and analysis. Manual image quantification for automated score validation was performed using FIJI/ImageJ (Schindelin et al., 2012).

**FIGURE 3:**
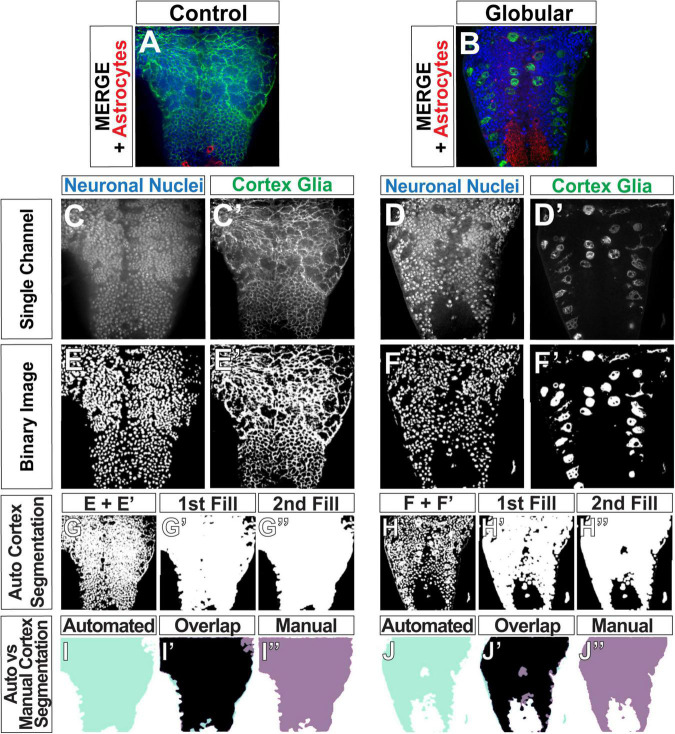
Automated determination of the cortex region. **(A,B)** Original images of control **(A)** and globular CG **(B)** consisting of three channels: CG (green), neuronal nuclei (blue), and astrocytes (red). **(C–F)** Gray scale images of single channels **(C–D’)** were converted binary images **(E–F’)**. **(G–H”)** Binary images of neuronal nuclei and CG were then combined to define the cortex **(G,H)**. Gaps between the nuclei and CG membranes were filled to produce solid white area covering the entire cortex **(G’,G”,H’,H”)**. **(I–J”)** Comparison of the cortex segmentations obtained by the automated pipeline (aqua, **I,J**) and by manual tracing of the same image (purple, **I”,J”**), with the overlap depicted between the two in black (**I’,J’**, black).

**FIGURE 4:**
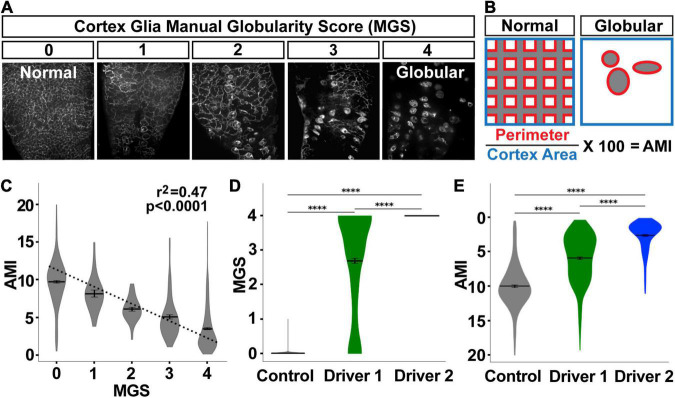
Automated morphology index (AMI) significantly correlates with manual scores for cortex glial globularity. **(A)** Cortex glial morphology was manually quantified using a 0–4 range, with 0 being normal mesh-like morphology, and 4 being almost completely globular (manual globularity score, MGS). **(B)** The automated morphology index (AMI) was calculated in the pipeline by measuring the perimeter of the cortex glia divided by the total area of the cortex. **(C–E)** AMI and MGS shows a significant negative correlation (**C**, *p* < 0.0001, *r*^2^ = 0.47, dotted line denotes the regression line). As globularity increases after RNAi knockdown using driver 1 (weaker) or driver 2 (stronger) **(D)**, the AMI decreases (**E**, inverted *y*-axis for ease of comparison to MGS). ****p < 0.0001.

**FIGURE 5:**
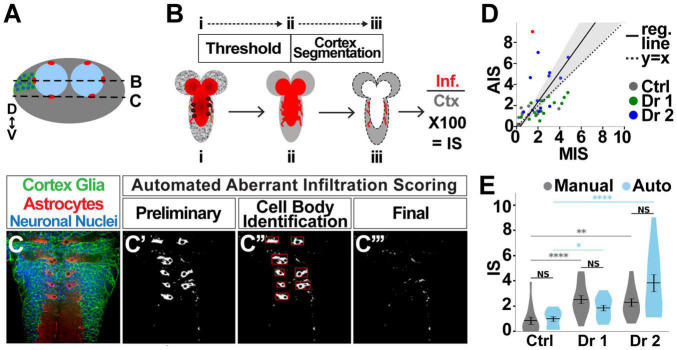
Automated quantification of astrocyte infiltration. **(A)** Cross section of the VNC depicting the location of astrocyte cell bodies, as well as the location of longitudinal sections shown in **(B,C)**. **(B)** Images were taken in longitudinal planes. The astrocyte channel was then thresholded, and the area of overlap between astrocytes and the cortex region was quantified as the infiltration score (IS). **(C)** Astrocyte cell bodies reside in the cortex under normal conditions **(C’)**, but were identified **(C”)** and excluded from the quantification to identify only aberrant cortex infiltration **(C”’)**. **(D)** Manual vs. automatic (auto) scores significantly correlate (95% confidence interval depicted by gray shaded area), with control (gray), driver 1 (green), and driver 2 (blue), (slope = 1.424, 95% CI [1.076, 1.772], *r*^2^ = 0.22, *p* < 0.0001, *n* = 42). Only one point was identified with a difference of greater than 6 (red) between manual and automated scores. **(E)** There is no significant difference between manual and automated scores for any of the groups. Pairwise comparison using Kruskal–Wallis test. NS *p* > 0.5 (control *p* = 0.343, driver 1 *p* = 0.678, driver 2 *p* = 0.155). *p* < 0.001 for Kruskal–Wallis test comparing three groups with same scoring methodology, followed by Dunn *post hoc* pairwise comparisons. *p < 0.05, **p < 0.01, ****p < 0.001. Control *n* = 14, KD driver 1 *n* = 15, KD driver 2 *n* = 13.

Denoising removes noise generated by factors such as light scattering and signal attenuation, and aids in generating more accurate binary representations of the raw image (Supplementary Figure 1). Compared to the original image (Supplementary Figure 1A), unsharp masking with scaling amount 2 and radius 20 produced the optimal image to identify astrocyte processes (Supplementary Figure 1B, red outline), where the sharpened image is produced by scaling (multiplying by the scaling amount) the difference between the original image and an image generated by adding noise in a radius-parameter-defined distribution (Malin, 1977). The same parameters were used for denoising the cortex glial channel, and the neuronal channel parameters were set to 3 and 20 for the scaling and radius, respectively. Other parameter values either failed to identify all astrocyte processes (Supplementary Figure 1B) or added undue noise (Supplementary Figure 1C) that would interfere with infiltration analysis in further steps of the pipeline. Denoised images are only approximations; therefore, to ensure accurate scoring by our pipeline, we included validation steps comparing results obtained by manual quantification with those obtained in an automated fashion (Figures 3–5).

Denoised images were then subjected to thresholding to convert grayscale to binary images, with the algorithm-selection process performed separately for each channel. Local algorithms, which consider only a pixel’s nearest neighbors (Sezgin and Sankur, 2004), were eliminated as possible candidates due to their poor performance, as judged by a qualitative comparison of the original and binary image. Second, 68 denoised images of the channel in question were binarized with seven global thresholding algorithms, where the image as a whole is used to calculate a thresholding limit (Supplementary Figure 2A; Sezgin and Sankur, 2004). For each of the sample images, a visual comparison of the original and binary image was used to determine the top three algorithms. The top algorithm was assigned a score of 3, the second-place algorithm was assigned a score of 2, and the third was assigned a score of 1. All other algorithms received a score of 0, and ties in this scoring were allowed. The final score for each algorithm was the sum of the scores for all the sample images. No single thresholding algorithm yielded satisfactory results for 100% of the sample images. We therefore implemented a decision tree into our pipeline to choose among the best thresholding algorithms for each channel in each image. We classified unsatisfactory results as those producing blown out images that contained superfluous signal or blacked out images where true signal was removed (Supplementary Figure 2B). First, the pipeline determines whether the image produced by Otsu’s method (Otsu, 1979) is blown out by exploiting the difference in texture between noise and true signal. Neuronal cell bodies have the appearance of distinct circles. Noise has the appearance of smaller, more densely and evenly distributed specks. Additionally, in blown out images, the majority of the noise being misrepresented as true signal was located in approximately the middle third of the image, the section corresponding to the neuropil. We converted all contiguous white objects smaller than 75 pixels to black, effectively removing specks, and the middle third of the image was then compared before and after despeckling. If despeckling resulted in a 7% or greater reduction in the number of white pixels, the image was determined to be blown out, and the Triangle algorithm (Zack et al., 1977) was used to threshold the image. Conversely, if the pipeline assessed that the binary image was blacked out, i.e., less than 2% of pixels were white, the pipeline will choose the Li thresholding algorithm (Li and Lee, 1993; Li and Tam, 1998) instead of Otsu’s method. A similar process was used to implement decision trees for selecting thresholding algorithms for the cortex glia and astrocyte channels. For cortex glia, the pipeline chooses between the Otsu and Triangle algorithms. For astrocytes, the pipeline chooses between the Otsu, Triangle and Yen (Yen et al., 1995) algorithms.

After the pipeline generates binary images for each channel (Figures 3E,F) using its selection of optimal algorithms, we perform a segmentation, or detection of the cortex by combining the neuronal nuclei and cortex glia binary images and adjusting the result (Figures 3G,H). Merely combining the two channels results in a cortex segmentation that is not completely solid (Figures 3G’,H’) due to gaps between the visualized neuronal nuclei and the cortex glia membrane, representing the rest of the neuronal cell body and cytoplasm. Since aberrant infiltration is quantified as the overlap between the cortex and astrocytes, a cortex segmentation with these gaps is likely to result in an undercount of infiltration. Hence, segmentation of the cortex was performed in four steps: merge (Figures 3G,H), 1st fill (Figures 3G’,H’), 2nd fill (Figures 3G”,H”) and a final finetuning step. The sequential fills were executed by our implementation of a dilation algorithm (Haralick et al., 1987), in which a black pixel was turned white if >10% of the neighbors within a 15-pixel radius were white, followed by Scikit-image’s remove_small_holes function (Walt et al., 2014), which turns to black any contiguous white objects smaller than set radius (5,000 pixels in our pipeline). Since the resulting segmentation was slightly larger than the true cortex region, an erosion algorithm—where a white pixel was turned to black if any of its immediate neighbors was black (Haralick et al., 1987)—was used to reduce the size of the segmented area.

### Automated Cortex Segmentation Validation

The functions used for automatically segmenting the cortex required a total of four parameters to be set: neighborhood (n) and threshold for the first fill step (thresh), hole size (hole_size) for the second fill, and the number of erosions (erosions) to finetune the segmented region size. In order to optimize values for these parameters, we compared the cortex region segmented in an automated fashion (automated, light green) to those produced manually (manual, purple) (Figures 3I,J). We used two metrics to determine the accuracy of the pipeline (Supplementary Figure 3): overlap (OL) divided by the manually segmented region (OL/M) and OL divided by the automatically detected (A) cortex (OL/A). A high OL/M score indicates that the pipeline is capturing a high percentage of the manually segmented region, interpreted as a high true positive rate. A high OL/A score indicates that the pipeline is not erroneously capturing areas that were not part of the manual segmentation, and can be thought of as low false positive rate. We used 42 images to test 192 different combinations of the four ROI-selection parameters (3–4 values per parameter, Supplementary Figure 3A). We chose the parameter set with the highest OL/M and OL/A scores (Supplementary Figure 3B, *n* = 15, thresh = 0.10, hole_size = 5000, erosions = 10, mean OL/M = 88.21%, mean OL/A = 85.46%). OL/M and OL/A scores of 100% would indicate a perfect overlap between the manual and automated ROIs. We would expect some small amount of error in the manual scores due to difficulty tracing a perfect outline of the cortex (given noise in images, limitations in image resolution, etc.). The majority of the scores lie above the mean, indicative of a highly accurate automated cortex detection method.

## Results

### Quantification of Cortex Glial Morphology

While cortex glial morphology is altered upon the loss of Spz3 or αSNAP, there can be variation in the degree of cortex glial globularity after genetic manipulation (Figure 4A). We automated the scoring of cortex glia morphology by utilizing the perimeter of the cells as a proxy for globularity (Figure 4B). As cells become more globular, there is a drastic reduction in their perimeter. The automated morphology index (AMI) for each image is calculated as the total perimeter of the cells, normalized by the total area of the cortex, and expressed as a percentage of that area.

As with cortex segmentation validation, we assessed AMI accuracy by comparing automated scores with manually obtained scores for the same images. For manual scoring, cortex glia images were assigned a manual globularity score (MGS) of 0–4 (Figure 4A). AMI and MGS are significantly negatively correlated (Figure 4C, *p* < 0.0001, *r*^2^ = 0.47), where a lower AMI indicates a greater morphological change. MGS was validated by comparing scores generated by three different blinded researchers for the same images, and finding a very high level of agreement between the scores as calculated by the intraclass correlation coefficient [249 images: ICC(3,k) = 0.983, *p* < 0.0001, Supplementary Figure 4]. When MGS scores were sorted by experimental group, the same patterns were revealed by MGS (Figure 4D) and AMI (Figure 4E, *y*-axis flipped for ease of comparison to Figure 4D). The no-RNAi control condition exhibited the lowest cortex glia globularity, indicative of normal morphology, followed by a LexA-driven knockdown of Spz3 (driver 1), and then a Gal4-driven knockdown of αSNAP (driver 2) with the highest globularity scores. These drivers specifically allowed us to explore variable morphological changes, as driver 1 is weaker than driver 2, which exhibited the highest variation in morphology. All pairwise comparisons of the groups are highly significant for both the manual and automated scores (*p* < 0.001, pairwise *post hoc* Dunn’s test), indicating that the pipeline is accurate in quantifying morphology.

### Quantification of Infiltration by Astrocyte Processes

Once the cortex has been segmented, the overlap between the cortex and astrocyte channel is measured in pixels and expressed as a percent of the total cortex area (Figures 5A,B). Astrocyte morphology introduces a complicating factor in quantifying infiltration, as astrocyte cell bodies are located within the cortex on the edge between the cortex and neuropil, as indicated by the circles in Figure 5A (red) and Figure 5B (dark red). However, these cell bodies do not constitute aberrant infiltration, and therefore need to be removed from the pipeline data to be quantified (outlined in red squares in Figure 5C’). Using the ratio of foreground to background pixels within the box bounding an object (Shi et al., 2009), the roundness of an object (Liukkonen et al., 2015), and area, we defined and excluded objects that are cell bodies while preserving aberrant infiltration (Figures 5C–C”’).

As with automating the morphological assessment of cortex glia, validating automated astrocyte infiltration scores is a critical step. By comparing 43 images using both methods, we found a highly significant correlation (*p* < 0.0001, *r*^2^ = 0.22) between the manual and automated infiltration scores (MIS and AIS, respectively). A correlation between perfectly matching sets of scores would be indicated by a slope of 1 (Figure 5D dotted line). We found an AIM vs. AIS correlation with slope = 1.424 95% CI [1.076, 1.772] (Figure 5D, solid black line surrounded by shaded gray region). There is a single case in which there was a discrepancy between the manual and automated scores of ±6 (red dot in Figure 5D). Without this point, which represents only 2.22% of the images considered in this validation procedure, the correlation slope is 1.19 95% CI [0.933, 1.452]. As part of AIS validation, we also examined automated scores for systematic errors in scoring, and found there is no experimental group for which the points lie solely on one side of the regression line (Figure 5D). Furthermore, scores pooled per experimental group and quantification method (Figure 5E) show no significant differences between AIS and MIS (pairwise Kruskal–Wallis comparisons, control *p* = 0.343, driver 1 *p* = 0.678, driver 2 *p* = 0.155). Finally, the two scoring methodologies indicate the same shifts in aberrant infiltration. Controls have the lowest infiltration, driver 1 shows intermediate infiltration, and driver 2 exhibits the greatest infiltration. Significant differences between scores obtained using the same methodology are color-coded: gray for manual scoring, blue for automated scoring (Kruskal–Wallis test followed by Dunn pairwise comparisons. *p < 0.05, **p < 0.01, ***p < 0.001). Taken together these data demonstrate that infiltration scores using the automated pipeline are accurate.

### The Relationship Between Cortex Glial Morphology and Aberrant Astrocyte Infiltration

**FIGURE 6:**
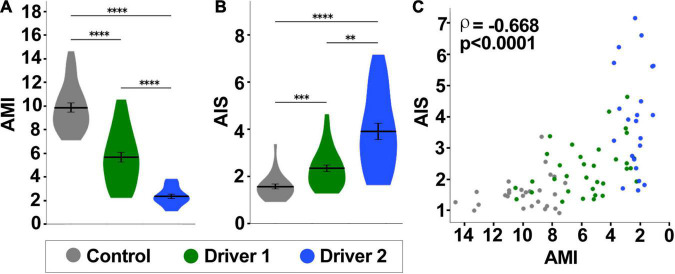
Astrocyte infiltration significantly correlates with disrupted cortex glial morphology. **(A,B)** As cortex glial (CG) morphology becomes more impaired, shown by reduced automated morphology index (AMI, in **A**), the automated astrocyte infiltration score (AIS) increases **(B)**. Kruskal–Wallis test comparing all groups, followed by Dunn *post hoc* pairwise comparisons. **p < 0.01, ***p < 0.001, ****p < 0.0001. **(C)** AIS and AMI are significantly correlated, as indicated by Spearman’s rank correlation coefficient (ρ = –0.668, *p* < 0.0001). Scores for individual images of each CNS were grouped and averaged by animal. Control *n* = 27, Driver 1 *n* = 34, Driver 2 *n* = 22 animals.

**FIGURE 7:**
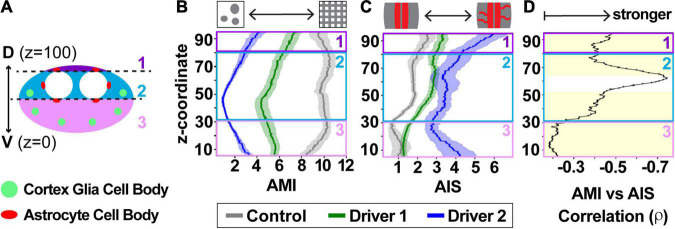
Cortex glia morphology, aberrant astrocyte infiltration, and their relationship vary along the dorsal-ventral axis of the ventral nerve cord. **(A)** Cross-section depicting the larval VNC along dorsal-ventral axis, divided into three zones: above the dorsal astrocyte cell bodies (1, purple), along the neuropil (2, teal), and ventral to the neuropil (3, pink). The locations of astrocyte cell bodies are shown in red, and the stereotyped location of globular cortex glia in green. **(B,C)** A sliding window reveals differences along the dorsal-ventral axis in both CG AMI **(B)** and astrocyte AIS **(C)**, with control (gray), driver 1 (green), and driver 2 (blue) depicted as mean ± SEM. **(D)** The relationship between AMI and AIS is shown along the dorsal-ventral axis as a black line indicating the Spearman’s rank correlation coefficient (ρ) for any given coordinate. The yellow shaded area indicates regions where the correlation was significant between the two with *p* < 0.001.

Scores for all images in the CNS were averaged to determine global scores for each animal for AMI (Figure 6A) and AIS (Figure 6B). These global scores were used in assessing the relationship between cortex glial morphology and astrocyte infiltration (Figure 6C). Correlations between AMI and AIS scores were assessed using Spearman’s rank correlation coefficient to allow for the possibility of a non-linear relationship between cortex glial globularity and aberrant astrocyte infiltration. Interestingly, not all areas of the CNS appeared to be equally affected, with apparent heterogeneity in both AMI and AIS along the dorsoventral axis. In order to explore the possibility of location-dependent heterogeneity in morphology and infiltration, we divided the CNS into three zones along the dorsal-ventral axis (Figure 7A), with the ventral surface set to a z-coordinate of 0 and the dorsal surface to 100. Differences in AMI and AIS were explored using a sliding window to analyze 10% of the total CNS depth at a time (Figures 7B,C). Scores for all images within each 10% window belonging to a single stack were averaged to produce a local dorsal-ventral depth score represented as mean ± SEM. AMI was higher throughout the CNS in the controls compared to either driver knockdown condition (*p* < 0.0001 for Kruskal–Wallis test comparing all groups, ****p < 0.0001, ***p < 0.001 with Dunn’s pairwise comparisons). AMI was lowest in the middle of the VNC, at approximately 40–50% along the dorsal-ventral axis. Average AIS was also higher throughout the VNC for knockdown animals; however, the inter-group difference in AIS was less pronounced than that of AMI. Driver 1 showed little to no difference in infiltration scores in the ventral 20% of the VNC. Additionally, there was only a modest increase in infiltration in the top 10–20% nearest the dorsal surface for animals in the driver 1 group. The largest increase in infiltration scores for these animals was found in the middle 50–80% of the VNC. In contrast, driver 2 showed the largest increase in infiltration near the surface of the VNC, at both ventral and dorsal ends, with a more subtle increase in the middle of the VNC. The correlation between local AMI and AIS was calculated using Spearman’s rank correlation coefficient (Figure 7D, ρ= −0.668, *p* < 0.0001). As cortex glial globularity increases, shown by a lower AMI, astrocyte infiltration correspondingly increases, indicated by the strong negative correlation. Significant correlations (*p* < 0.001) among all groups are indicated by the yellow shaded region, which occurs throughout the dorsal-ventral axis with the exception of a small section in the middle of the VNC (52–64%, *p* > 0.05 from 55 to 60%).

## Discussion

Glial tiling is a phenomenon that occurs throughout the animal kingdom (Oberheim et al., 2009; Hughes et al., 2013; Kettenmann et al., 2013; Stork et al., 2014; Nichols et al., 2018), yet we still know relatively little about the formation, maintenance, and function of glial domains. While human astrocytes do still assemble into tiled domains, the extent of overlap of their processes is higher than in rodents (Oberheim et al., 2009), and astrocyte territories in species such as in ferrets can exhibit as much as 50% overlap in some astrocyte populations (López-Hidalgo et al., 2016). Interestingly, these tiling domains can also vary within the same species, such as in disease states like epilepsy (Oberheim et al., 2008) or even during normal aging (Grosche et al., 2013). Protoplasmic astrocytes in 5 month old adult mice show little to no overlap in their domains, yet the overlap increases in both cortical and hippocampal astrocytes nearly two-fold by 21 months of age (Grosche et al., 2013). Exactly what molecular cues regulate glial domain tiling and organization, and how and why these change in aging or disease is currently unclear.

We and others have shown that *Drosophila* present an exciting model to study the molecular underpinnings of glial tiling between either the same or disparate subtypes of glial cells. Rodent models that rely on genetic labeling of glial cells with Cre lines (Hassala et al., 2007; Degen et al., 2012; Srinivasan et al., 2016) often lack precise single cell genetic manipulation and differential control of adjacent cells. *Drosophila* provide genetic tools to easily target, label, and manipulate single cells within the same subtype (Figures 1B–F), as well as multiple different glial subtypes simultaneously (Figures 1G–J), with a plethora of publicly available tools for genome-wide manipulation. Additionally, the domain organization of the *Drosophila* CNS allows for investigations of glial-glial tiling interactions that would be much more difficult in other organisms (Figure 1A). Previous approaches have been applied to murine astrocytes to simplify the quantification of astrocyte tiling, such as using the volume of sparsely labeled astrocytes by Golgi impregnation, and taking into account the total astrocyte number in a given tissue area (Grosche et al., 2013). Interestingly, these authors found that in young mice, the overlap ratio of astrocytes was below 1, suggesting close interactions of other glial subtypes. Our previous findings that astrocytes react to cortex glial dysfunction by crossing the neuropil-cortex boundary (Coutinho-Budd et al., 2017) provided a basis for our current line of research. We wanted to build upon these findings to understand the extent of astrocyte reactivity when cortex glial morphology and tiling were disrupted; however, manual quantification of glial tiling is cumbersome, unfeasible on a large scale, and could miss more subtle differences in tiling variation. Here we have presented and validated an automated pipeline using free, open-source software to quantify both glial morphology and domain infiltration of adjacent glial subtypes in *Drosophila.* This tool allows for high-throughput quantification that, when combined with the power of genetics in this model system, will open the door for large scale, *in vivo* mechanistic studies of glial tiling.

The formation of globular cortex glia would be expected to leave neuronal cell bodies without any glial contact; however, the ability for glial cells to grow is quite impressive, and the surrounding healthy glia do not leave those neurons bare for long. Our previous work identified that upon the loss of Spz3 or αSNAP in cortex glia, thin astrocytic processes began to move into the cortex in late larval stages (Coutinho-Budd et al., 2017). We have created and validated a pipeline to automatically quantify both cortex glia morphology (AMI) and aberrant astrocyte infiltration into the cortex (AIS) for over 3,300 images, with accuracy confirmed by comparison with manually obtained scores. Using this automated pipeline, we found that the extent of astrocyte infiltration strongly correlates with the extent of cortex glial disruption, but importantly, that this correlation remains regardless of the high degree of variation in both categories throughout the CNS. The observed variation could result from a number of different factors, including but not limited to glial heterogeneity, location and positioning of glial cells throughout the CNS, and driver strength. Glial heterogeneity is a current focus within the glial field to understand how different cells even within the same subtype (i.e., astrocytes) differ in their molecular composition and functional roles. With the recent advancement of single-cell sequencing technologies (Klein et al., 2015; Macosko et al., 2015), cellular heterogeneity is becoming more widely understood beyond simple morphological differences such as fibrous or protoplasmic astrocytes. Different glial cells of the same subtype within a defined brain region can even exhibit molecular variation in signaling factors, receptors, transcription factors, and more (Khakh and Deneen, 2019; Li et al., 2019; Hasel et al., 2021), meaning that the same genetic perturbations in or near two adjacent cells could produce two very different reactions. While the extent of heterogeneity within each *Drosophila* glial subtype is thought to be less than that of mammalian glia, differences within the same subtype have been noted, such as the higher distribution of fatty acid binding protein (fabp) and lipid droplets in superficial cortex glia (Kis et al., 2015). In the current study, we made use of two different driver systems: driver 1 was used to knock down Spz3 with the LexA system, resulting in a wider range of disrupted cortex glial morphology compared to the stronger driver 2, which uses the Gal4 system to knock down αSNAP. These differences in strength allowed us to investigate how astrocytes react to mild and more severe perturbations in glial tiling and boundary maintenance. Notably, the severity in AMI is more likely to be due to the strength of the driver rather than the molecule knocked down, as the Gal4-driven knockdown of Spz3 results in a similarly severe morphological disruption as Gal4-driven knockdown of αSNAP (Coutinho-Budd et al., 2017); however, we cannot definitively rule out molecular differences, as the reduction of αSNAP could lead to restricted release of other secreted signaling factors. The differences in the degree of cortex glial disruption with driver 1, regardless of the underlying mechanism, allowed us to address the significant correlation between the extent of the globular morphology and aberrant astrocyte outgrowth, a result that was further supported with the stronger driver system.

The layout of the CNS is not homogenous throughout the dorsal-ventral axis. The neuropil is offset toward the dorsal side of the VNC (Figure 1A’), with many astrocyte cell bodies distributed throughout this region. The ventral region of the VNC contains more cortex glia and neuronal cell bodies (zone 3, Figure 7A), located farther from the neuropil where astrocyte processes reside. Therefore, if an astrocyte infiltration signal originates from cells within the ventral cortex, the signal could take longer to reach the astrocytes or never reach it at all. Alternatively, there could be a larger signal arising from the greater number of neuronal cell bodies within this region. Likewise, there is more space for astrocytes to grow in this direction. The combination of these factors complicates the investigation of glial tiling, but the ability to automate quantification throughout the VNC allowed us to reveal differences in reactivity and correlation in spatial segregation along the dorsal-ventral axis that would have been difficult to identify via manual quantification alone, and to begin to parse out the cellular reactivity.

In addition to the dorsal alignment of the neuropil, the location of both astrocyte and cortex glial cell bodies is a potential source of AMI and AIS variation along the dorsal-ventral axis. While control *Drosophila* can have up to an average of 60–80 cortex glial nuclei in the thoracic segments of the VNC, those with globular cortex glia average 6–10 nuclei per segment due to a failure to proliferate (Coutinho-Budd et al., 2017). Moreover, these remaining globular cortex glial cells are located in stereotyped positions from animal to animal at the location where the cortex glial nuclei first align during development (Beckervordersandforth et al., 2008; Coutinho-Budd et al., 2017). This spacing leaves a greater distance between the neuropil and the ventral cortex glial cells (zone 3 in Figure 7A) compared to those located in the more lateral position in the middle of the dorsal-ventral axis (zone 2 in Figure 7A), and also allows for more variability if not all of the cortex glial cells are fully transformed from the mesh-like to globular morphology. Interestingly, the dorsal-most neurons of the VNC (zone 1, Figure 7A) are encapsulated by cortex glia in the lateral portions, but a specialized type of ensheathing glia wrap the more medial neurons in this portion of the VNC (Coutinho-Budd et al., 2017; Pogodalla et al., 2021). This could account for the greater variation in zone 1, along with the intriguing possibility that cortex glial dysfunction disrupts other adjacent glial subtypes beyond astrocytes. While it is clear that the aberrant infiltration is due to astrocytes extending processes into the cortex rather than a migration of the entire cell (Coutinho-Budd et al., 2017), astrocyte cell bodies do reside in the cortex along the interface of the cortex and neuropil (Stork et al., 2014). In order to quantify only those processes that account for true aberrant infiltration, we identified characteristics of cell bodies that would allow for their automatic exclusion from the final infiltration count. However, slight remnants left behind from the sheer number of astrocyte cell bodies, could be artificially increasing infiltration scores in regions surrounding the neuropil (zones 1 and 2, Figure 7A).

We found that AMI and AIS strongly correlate throughout the majority of the VNC, though our analysis revealed that cortex glial morphology, astrocyte infiltration, and their relationship varies along the dorsal-ventral axis. Moreover, there was still variation in AIS amongst different animals or regions even with strong disruption of cortex glial morphology. While the automated pipeline presented here is designed for general use for anyone with access to simple imaging methods like confocal microscopy, one limitation is that glial domains are three-dimensional structures, and two-dimensional imaging can miss fine processes between the imaging intervals that could lead to an underestimation of glial territory and infiltration. Future development of a pipeline to work with three-dimensional imaging software and imaging techniques such as light sheet microscopy will further enhance these studies.

Identifying the molecular mechanisms that underly the development and maintenance of glial boundaries, and how and why glial cells respond to move out of their normal territories, as well as the functional consequences of doing so is paramount to furthering our understanding of the nervous system in health and disease. We now have a strong genetically tractable system to investigate these issues with an optimized tool for quantifying and revealing changes in glial tiling. This work raises a number of intriguing questions that we can use these tools to begin to answer: what are the molecular mechanisms involved in setting up and/or maintaining glial tiling? How do they change in aging or disease? Do glial tiling cues result only from glial-glial interactions or neuron-glial communication as well? Finally, if glia divert their cellular resources from their normal positions, such as astrocytes from the neuropil into the cortex, can they still maintain their normal functions? The combination of the automated pipeline provided here with the plethora of genetic tools available in *Drosophila* will allow us to begin to unlock the answers.

## Data Availability Statement

The datasets presented in this study can be found in online repositories: https://github.com/gabys2006/TilingGlia, with test images available at http://cellimagelibrary.org/groups/54646.

## Author Contributions

JC-B and GS conceived and designed the study and prepared the manuscript. GS performed the animal experiments, image acquisition, design of the pipeline, and statistical analysis. GS, GR, and AM performed blinded manual quantifications for validation of the automated data. All authors contributed to the article and approved the submitted version.

## Conflict of Interest

The authors declare that the research was conducted in the absence of any commercial or financial relationships that could be construed as a potential conflict of interest.

## Publisher’s Note

All claims expressed in this article are solely those of the authors and do not necessarily represent those of their affiliated organizations, or those of the publisher, the editors and the reviewers. Any product that may be evaluated in this article, or claim that may be made by its manufacturer, is not guaranteed or endorsed by the publisher.
