# Supplementary material for: Quantifying Glial-Glial Tiling Using Automated Image Analysis in Drosophila
Source: Front Cell Neurosci. 2022 Mar 24;16:826483. doi: 10.3389/fncel.2022.826483 (PMC8987577; doi:10.3389/fncel.2022.826483)
Supplement: Supplementary file 1 [file Data_Sheet_1.pdf]

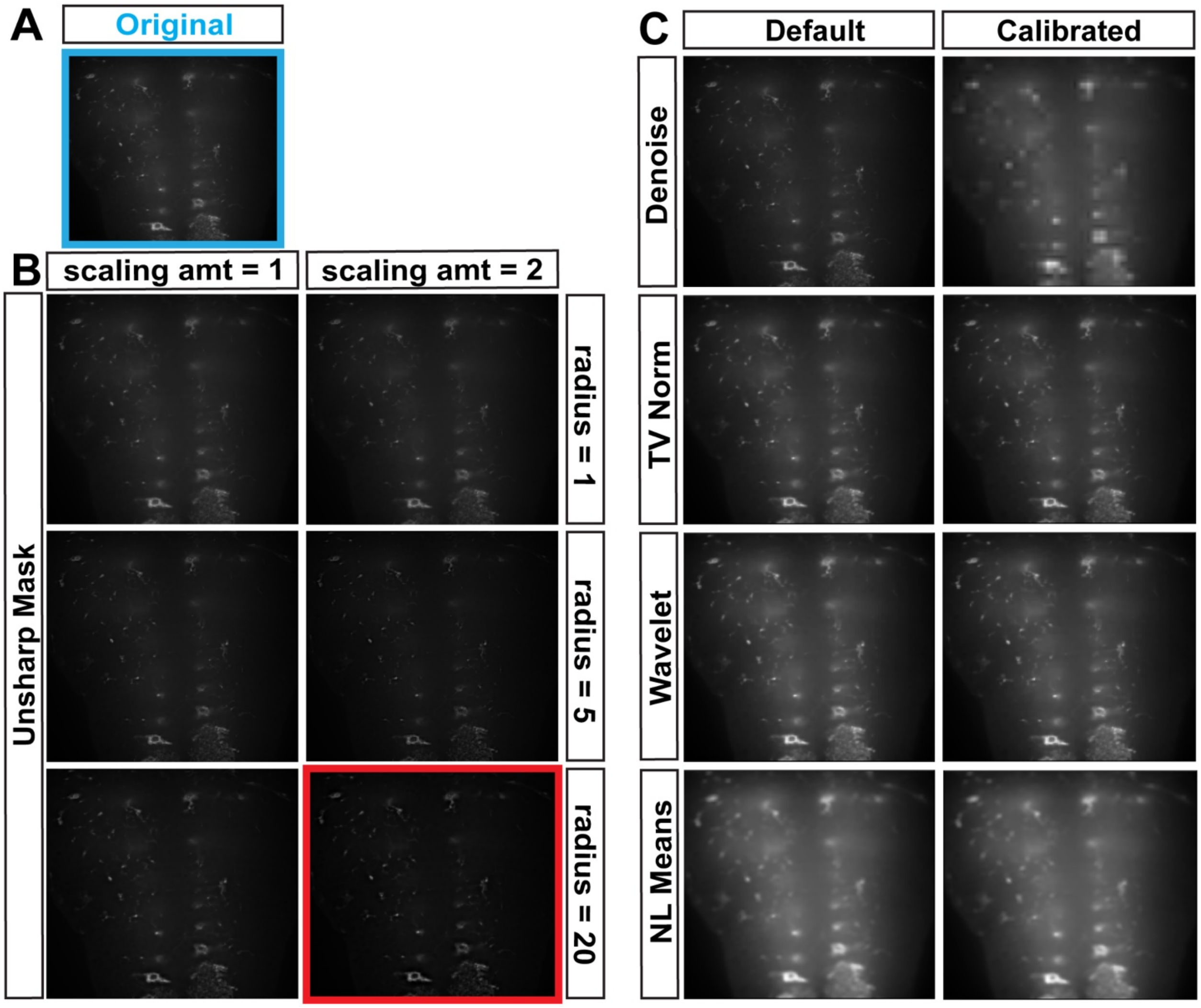

Supplemental Figure 1. Examples of denoising algorithms. A) The original astrocyte image (blue outline) was denoised using different algorithms implement by scikit-image. **B)** Six panels resulting from denoising using an unsharp mask with different combinations of parameters, radius of 1, 5, or 20 and scaling amount (amt) 1 and 2. The red outline indicates the algorithm selected for denoising astrocyte images, as it produced the truest match of the original image. **C)** Eight panels resulting from denoising with denoise, non-local (NL), wavelet, and total-variation (TV) algorithms using two parameter combinations per algorithm: default and optimized using a J-invariant algorithm (calibrated).

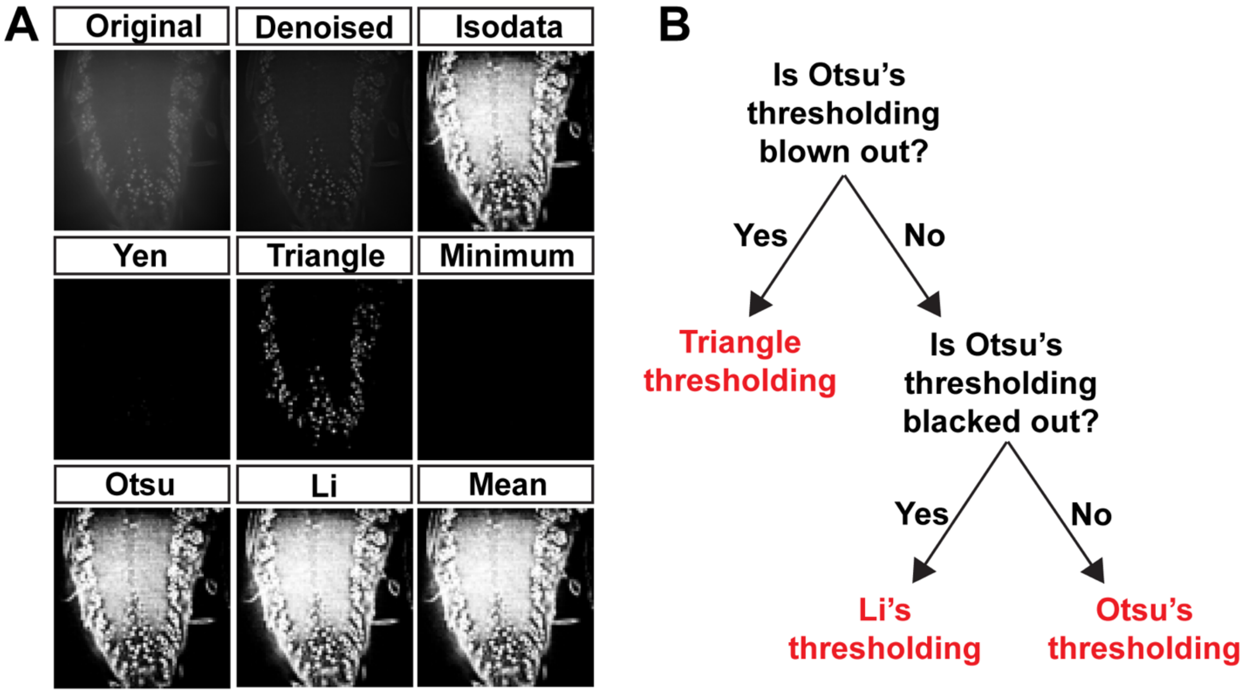

Supplemental Figure 2. Examples of binarization algorithms. A) The original neuronal cell nuclear image was denoised and binarized by 7 global thresholding algorithms implemented by scikit-image. **B**) Decision tree used to determine the thresholding algorithm for each neuron image. Blown out images vastly overestimated the area covered by neurons, such as those produced by Isodata, Otsu, Li, and Mean algorithms in (A). Blacked out images are those that vastly underestimate the area covered by neurons, such as those produced by Minimum and Yen algorithms in (A). Similar decision trees were used to produce binary images for the cortex glia and astrocyte channels.

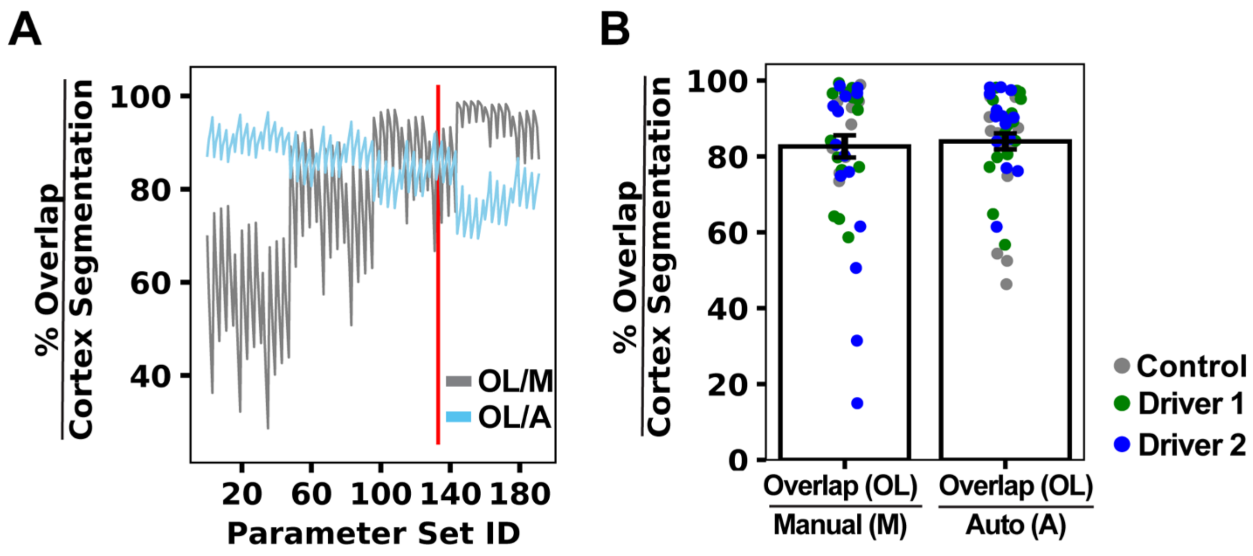

Supplemental Figure 3. Optimization and validation of automated cortex detection. A) Automated cortex segmentation was produced for 42 images using 192 different parameter sets. The overlap (OL) between corresponding automated and manual segmentation was calculated. The average OL/manual ROI (OL/M) and OL/automated (OL/A) were used as performance metrics to select the best parameter combination (red line) for subsequent analyses. **B**) OL/Cortex Segmentation scores for the top-performing parameter combination for the 45 validation images (control: gray, driver 1: green, driver 2: blue).

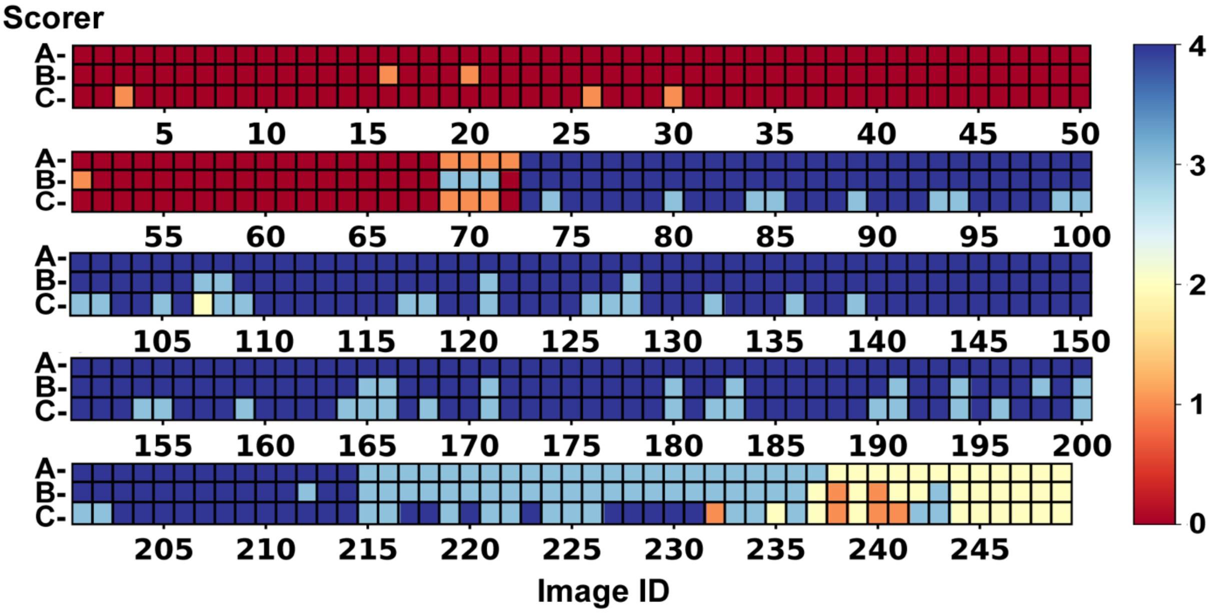

Supplemental Figure 4. Validation of the manual globularity score, and the ruling out of quantifier variability. Heatmap illustrating the manual globularity score (MGS) for 249 images as determined by three blinded quantifiers. There is a high level of agreement between the scorers as indicated by the intraclass correlation (ICC(3,k)=0.983, p<0.0001).
